# Community perceptions of mental health needs: a qualitative study in the Solomon Islands

**DOI:** 10.1186/1752-4458-3-6

**Published:** 2009-03-13

**Authors:** Ilse Blignault, Anne Bunde-Birouste, Jan Ritchie, Derrick Silove, Anthony B Zwi

**Affiliations:** 1 School of Public Health and Community Medicine, Faculty of Medicine, The University of New South Wales, Sydney, Australia; 2 School of Psychiatry, Faculty of Medicine, The University of New South Wales, Sydney, Australia

## Abstract

**Background:**

Psychosocial and mental health needs in the aftermath of conflict and disaster have attracted substantial attention. In the Solomon Islands, the conceptualisation of mental health, for several decades regarded by policy makers as primarily a health issue, has broadened and been incorporated into the national development and social policy agendas, reflecting recognition of the impact of conflict and rapid social change on the psychosocial wellbeing of the community as a whole. We sought to understand how mental health and psychosocial wellbeing were seen at the community level, the extent to which these issues were identified as being associated with periods of 'tension', violence and instability, and the availability of traditional approaches and Ministry of Health services to address these problems.

**Methods:**

This article reports the findings of qualitative research conducted in a rural district on the island of Guadalcanal in the Solomon Islands. Key informant interviews were conducted with community leaders, and focus groups were held with women, men and young people. Wellbeing was defined broadly.

**Results:**

Problems of common concern included excessive alcohol and marijuana use, interpersonal violence and abuse, teenage pregnancy, and lack of respect and cooperation. Troubled individuals and their families sought help for mental problems from various sources including chiefs, church leaders and traditional healers and, less often, trauma support workers, health clinic staff and police. Substance-related problems presented special challenges, as there were no traditional solutions at the individual or community level. Severe mental illness was also a challenge, with few aware that a community mental health service existed. Contrary to our expectations, conflict-related trauma was not identified as a major problem by the community who were more concerned about the economic and social sequelae of the conflict.

**Conclusion:**

Communities identify and are responding to a wide range of mental health challenges; the health system generally can do more to learn about how this is being done, and build more comprehensive services and policy on this foundation. The findings underscore the need to promote awareness of those services which are available, to extend mental health care beyond urban centres to rural villages where the majority of the population live, and to promote community input to policy so as to ensure that it 'fits' the context.

## Background

### Introduction

The landmark World Development Report of 1993 identified mental disorder as an important contributor to the Global Burden of Disease. Since then efforts have been made to ensure that mental health issues attract the necessary attention and resources to address this burden of ill-health and disability, concerns re-emphasised recently in a campaign mounted by the Lancet, the World Health Organization (WHO) and key players in the global health field. In 2007, the International Journal of Mental Health Systems highlighted the "urgent need to focus on the development of effective, appropriate, affordable and equitable mental health systems", arguing that a major impediment to the achievement of this goal is "the lack of evidence for what kinds of mental health systems are appropriate and effective in varying political, social and economic contexts"[1].

The Lancet[2] has highlighted the importance of building the evidence base around mental health and health services, drawing attention to the resources required for more effective and equitable mental health services provision[3]. Alongside this is a growing body of empirical research suggesting that prolonged conflict adds to the burden of mental disorder – a double jeopardy with the effects of under-development and conflict reinforcing one another; the Solomon Islands being a case in point[4].

Documentation of mental health policy and practice in low and middle-income countries and, in particular, the countries in the Pacific, is sparse. There is also a dearth of insights from post-conflict countries, especially in relation to mental health system development. In this paper we address aspects of this gap by presenting findings emerging from our study of psychosocial and mental health policies, from the bottom up, in the Solomon Islands. Relatively few studies have assessed perspectives on mental health and psychosocial wellbeing from the community level, attempting to draw links between the knowledge and perceptions of the community (what are the problems and what should be done) and services currently in place.

This paper reports a major component of one of our national case studies, conducted as part of a larger project seeking to identify how psychosocial wellbeing and mental health are conceived and addressed in relation to policy agenda-setting, formulation and implementation in two countries in the Asia-Pacific region – the Solomon Islands and Timor-Leste (East Timor). The focus of the paper is on community experience within the Solomon Islands, the first country participating in this research.

### Research approach

**Figure 1:**
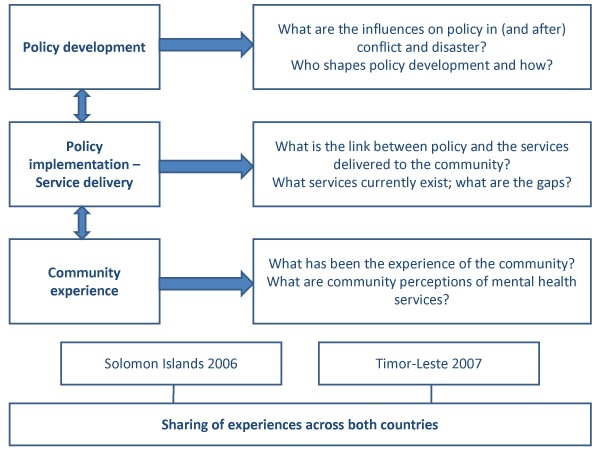
**Research approach**.

Our overall research approach is described in Figure 1. Research questions were framed according to three levels of the overall health system – policy, service delivery and community. The broader project will seek to clarify the links between these levels, guiding our data collection and analysis (Figure 1). Here we examine the community perspectives on mental health and psychosocial wellbeing, highlighting their importance in guiding service delivery and policy formulation.

### Country background

The Solomon Islands is a low-income country[5] located in the South-West Pacific. It comprises nearly one thousand islands with a land area of 304,000 km^2 ^spread over a sea area of about 1.5 million km^2^, making communication, travel, and service delivery difficult and creating inequities in access. The population of 0.5 million people is young, with 42% aged less than 15 years[6]. Melanesians comprise the dominant ethnic group (93%), and 98% of the population belong to a Christian church. The population is extremely diverse with some 91 indigenous languages and dialects being spoken in addition to Solomon Islands Pijin (the most common language) and English (the official national language). Over 83% of the population live in rural areas where subsistence agriculture, fishing, and food gathering are the main income source[7].

From 1998 to 2003, the Solomon Islands experienced a period of armed conflict known locally as the 'tensions', occurring primarily on the island of Guadalcanal where the capital, Honiara, is located. Contributing factors included competition for land and scarce resources, particularly between people from Guadalcanal and the neighbouring island of Malaita. Despite the signing of the Townsville Peace Agreement in October 2000, hostilities continued and escalated between rival groups in southern Guadalcanal. Peace and security were eventually restored in 2003 with the arrival of the Australian-led multinational Regional Assistance Mission to the Solomon Islands (RAMSI). The conflict resulted in 150–200 deaths and over 35,000 people were displaced internally throughout Guadalcanal and Malaita[8].

Changes in the social and economic environment, accelerated by the conflict, appear to have had an adverse impact on mental health, both in terms of access to services and as a consequence of stress and social disruptions[9]. A national consultation conducted in 2005 for the Solomon Islands Ministry of Health found widespread concern about psychiatric illness (including functional and organic psychosis, bipolar disorder, severe depression and anxiety, and post-traumatic stress disorder) substance abuse and psychosocial problems[10].

Within the Ministry, mental health is one of three community-based programs, the other two being social welfare and community-based rehabilitation (with a focus on physical disability). Some 1.4% of the total health budget is directed to mental health[11]. There is no national substance abuse program. The churches and local and international non-government organisations (NGOs) deliver a range of welfare services and psychosocial interventions to women, youth and families including counselling and community development. None of the NGOs focus specifically on providing core mental health services. However, there is a trauma support program (run by a faith-based NGO with funding from the Australian Agency for International Development – AusAID) that offers counselling for people who are suffering from stress and trauma.

Understanding patterns of help seeking and pathways to care is essential to developing effective mental health policy and services. The Solomon Islands Government has recognised the challenges it faces across the health service as a whole. The National Health Strategic Plan 2006–2010 noted that "utilisation of health care is on the increase, but communities make decisions in a society that still utilises home care with traditional healers and western medicine"[10]. There is little documentation on community approaches to mental disorders and psychosocial problems, particularly in rural areas, and scant research on what services should be offered, and how.

Choosing a rural district that was illustrative rather than exceptional, we sought to answer the following questions: How do people understand mental health and 'mental problems'? Where do they turn for help? What do they know about the mental health service? We judged that these were central questions to address in order for services and policies to respond effectively to the needs of communities that have experienced major periods of conflict and social upheaval.

## Methods

### Research team and advisors

Two of the researchers made up the in-country team for this component, an Australian female (IB) and a Solomon Islander male (AV). The latter came from the area studied, spoke the local languages, and possessed basic research skills that were actively nurtured and supported throughout the data collection and analysis stages. Additional input was offered by Ministry of Health staff, and advice on local issues and culturally appropriate ways of working was provided by a local Research Advisory Group. Guides for the key informant interviews and focus groups (English and Pijin) were developed collaboratively by IB and AV, with advice from the Research Advisory Group.

### Setting

The site for the community study was selected following extensive in-country consultation. We sought to identify a site that was typical rather than exceptional and that met the following criteria: outside the capital city (capital cities are usually very different from the rest of the country); links with both urban and rural communities; median level of access to services (relative to the rest of the country); affected by conflict; and presence of some international donor and NGO activity. The site chosen, on the island of Guadalcanal, met the criteria and had not previously received much attention from health or other researchers.

### Community engagement and recruitment

Contact with community leaders was facilitated through the provincial health service, the church and a local faith-based NGO that had a strong presence in the area. On initial visits, we introduced ourselves and explained the research to health clinic staff, church leaders and the school principal. In the 10 villages visited, we first approached the chief and sought his support and advice. Planned visits to the different villages were announced by the catechist at the local church the preceding Sunday. During a visit, people were approached individually and asked to participate in an interview or focus group. Plain-language information and consent forms were provided in both English and Pijin.

The research was carried out over four months from August to November 2006.

### Data collection

Data collection methods included mapping community resources, in-depth interviews, focus group discussions and participant observation.

Community mapping was undertaken as part of the general data collection and involved identification of the resources available (individuals, groups and organisations) based within or external to the community, and the relationships between them. In-depth, semi-structured interviews with community leaders covered their role in the community and their response to people with psychosocial and mental health problems, including involvement of other individuals, groups and/or services. Focus groups were designed to explore the community's perspectives on mental health issues and support services (government, non-government, church-based and traditional healers), and to assess their knowledge of mental health services. We asked about community responses to mental and psychosocial problems, and about local concepts of mental health and wellbeing, mental problems and mental illness. We also asked about the role of key actors and organisations in providing mental health care and/or in attempting to prevent mental disorders and psychosocial problems: family, kastom (custom) doctors and chiefs, churches, NGOs and health services. Respecting local sensitivities, separate focus groups were arranged for young men and young women (under 29 years) and for older men and older women (over 30).

**Figure 2:**
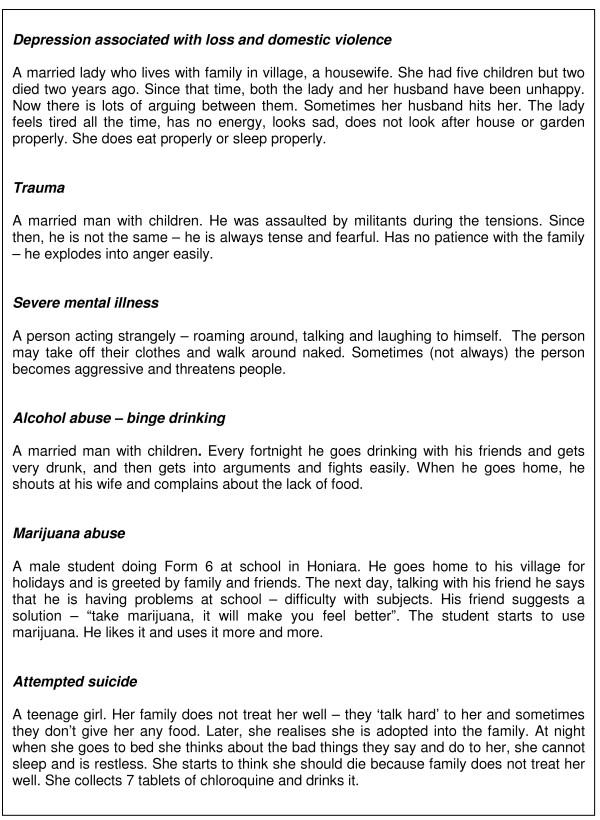
**Vignettes**.

We used vignettes to explore how members of this community responded to a range of mental and social problems: How do they perceive the problem? Where do they turn for help? What happens if treatment fails? How does the community assist people like this? Vignettes allow actions and occurrences to be explored within their context and provide a non-threatening way to investigate sensitive topics like mental disorders without using technical language[12]. We chose as our 'tracers', problems that had been identified as issues of concern in the policy component of this study: severe mental illness, post-traumatic stress disorder, depression associated with loss and domestic violence, alcohol abuse (binge drinking), marijuana abuse and attempted suicide. The final versions (Figure 2) were based on examples provided by mental health service staff and refined in field tests; at least four were introduced into each interview and focus group. If respondents volunteered a scenario of their own, the corresponding vignette was disregarded.

The local researcher spent up to week at a time in the area conducting interviews and focus groups, taking part in general community activities, and observing, alongside the formal data collection.

### Data management and analysis

Community mapping was documented in pictures and words, providing contextual insights for this component of the study. During interviews and focus groups, notes were taken. Most were also digitally recorded. Most of the English-language interviews were transcribed directly from the audio file except where the sound quality was poor, while notes from the other interviews and focus groups were translated and written up in English, with additions from the audio file when required. Transcripts were reviewed, corrected, and then coded for analysis using NVivo7. Interview, focus group and field note data were collated. We identified themes that emerged from these data as well as being responsive to the key issues identified for examination by the project and included in our interview questions.

### Ensuring rigour

Several techniques were employed to ensure the rigour of the research. Field notes were kept separately by each researcher, and regularly reviewed and discussed. Key informants were recruited to encourage a range of perspectives from across the area. We proposed in advance to run at least three focus groups with each of the four gender/age combinations, resulting in 12 groups in all. By the end of data collection, we reached a consensus that saturation had been reached, thus precluding the need to recruit further. Multiple methods of data collection and data sources enabled triangulation. Unusual or unexpected findings were explored further. Member checking was achieved through presentation of the findings at a community meeting held under a large rain tree after church.

### Ethics

Overall ethics approval was sought from the Solomon Islands Human Research Ethics Committee (HREC) and the University of New South Wales HREC. At the village level, general approval for consultation with community members was obtained from the local chiefs, prior to approaching individuals. At the community high school and college, approval was obtained first from the principals and then from participating individuals.

Given that our enquires could raise sensitive issues, we took steps to guard against emotional distress. Prior to the field work we put in place systems to support anyone who became distressed during an interview or focus group, or who was need of urgent mental health care. We also arranged for staff from the mental health service to accompany us on our initial visits. The community meeting was attended by both the Provincial Mental Health Program Officer for Guadalcanal and the Director of the Mental Health Service who gave a short presentation on the mental health service and let people know how they could obtain assistance for themselves or a family member. At that meeting we distributed plain-language summaries of the findings in both Pidgin and English, with the intention that they could be used as tools for community advocacy.

A national workshop was organised and co-facilitated with the Ministry of Health. We arranged for seven of our community informants to attend the workshop so that their experiences and opinions could be directly shared with policy-makers and service managers. A policy briefing paper was later prepared for the Ministry.

## Results

### Context

Our community mapping concentrated on the catchment area of the rural health clinic, which has a total population exceeding 1,500 people. A Catholic Mission had been established there in 1904. The mission station has a large church, a government primary and secondary school and a vocational college for girls, in addition to the government clinic. AusAID has funded road maintenance; however there has been little sustained NGO activity other than the trauma support program.

The location has road access to the capital, where people regularly market their vegetables. Villages stretch to the east and west of the mission station, between the hills and the coast. To the west there is another central church where people from surrounding villages worship, while the east has three small local churches. There were notable differences between villages within a relatively small geographical area, with villages to the west being more exposed to outside influence and those to the east being more traditional. The main source of income is growing food for market, copra, cocoa and betel nut. Many traditional norms, values and practices remain intact, for example around marriage and child-rearing. Guadalcanal is a matrilineal society and women are the customary land owners.

During the conflict, access to Honiara was completely cut off. There was shooting at the mission station (today a wooden cross in the grass marks the spot where a man was killed) and villages on the eastern side were burned in a raid.

### Local informants

We interviewed 23 community leaders: five church leaders, two women's group leaders, the nurse and a nurse aide from the health clinic, the chair of the high school board and two teachers, the principal and a teacher from the college, four chiefs, three kastom doctors and two trauma support workers. Twelve focus groups were conducted in eight locations with groups ranging in size from three to six participants, eliciting the views of 24 men and 29 women across a wide range of ages. Groups were held in a public place, usually a veranda or outside the church. At times other people came and listened.

The local researcher conducted nine of the interviews in Pijin, with occasional local language additions. The remainder (14) were conducted by IB, with informants using English and sometimes Pijin. AV conducted ten of the focus groups using Pijin and IB conducted two women's groups.

On our first visit people usually directed us to the chief or catechist. Both researchers easily engaged with these community leaders as the expected gatekeepers. The community members most difficult to engage were the young men, especially ex-combatants. With time, however, AV was able to gain their trust. Interestingly, young men from two villages chose to be interviewed together so each group could hear what the other was saying. Interviews were usually carried out before or after people returned from tending their gardens in the bush.

Recruiting females for interviews and focus groups proved a challenge for the male researcher, and required great cultural sensitivity. The fact that AV was known to the community also created challenges because people expected him to already know the answers to the questions and to be familiar with traditional practices, whereas a foreigner might be forgiven for being ignorant. In exploring traditional practices, kastom doctors were reluctant to disclose details of their techniques.

### Main community problems reported

Irrespective of location, community problems perceived as having an impact on the mental health and wellbeing of members were of growing concern. There were some differences in emphasis by social group and/or position in the community (Table 1).

**Table 1:** Main problems by community group

Problem*	**Community leaders**	**Older men**	**Older women**	**Young men**	**Young women**
**Excessive drinking/Drunkenness**	**X**	**X**	**X**	**X**	**X**

**Smoking marijuana**	**X**	**X**		**X**	**X**

**Antisocial behaviour – Swearing and shouting**	**X**	**X**	**X**	**X**	**X**

**Fighting**	**X**	**X**	**X**	**X**	**X**

Domestic violence	X	X	X		

Disagreements within the family	X	X	X		

Jealousy		X	X		X

Extramarital affairs		X		X	X

Broken families	X				

**Teenage pregnancy**		**X**	**X**	**X**	**X**

Children too close			X		

Children left to roam			X		

Single mothers					X

School dropouts					X

Students struggling at school	X		X		

Struggle to make a living	X		X		X

Land disputes	X	X			

Sexual assault	X		X		X

Sorcery	X		X		

Stress and worry	X		X		

**Lack of respect**	**X**	**X**	**X**		**X**

Lack of involvement in communal activities	X	X		X	

**Lack of cooperation**		**X**	**X**	**X**	**X**

Poor community leadership		X	X		X

Lack of government assistance	X				

People traumatised	X				

*Problems listed in bold indicate concerns common across the groups.

Problems identified by key informants and focus group participants. Additional problems raised at the community meeting included conflict between cultural practices and church teachings, young children drinking and smoking, stealing, child neglect, marital and family problems, and traditional enmities.

Overall, the problems given greatest priority were excessive drinking (store-bought alcohol, home brew and kwaso – distilled home brew), marijuana use, swearing and fighting, teenage pregnancy, lack of respect, and lack of cooperation. In most people's minds, excessive drinking and drunkenness were associated with fighting, swearing, domestic violence and extra-marital sex. Excessive marijuana use was considered a frequent cause of severe mental illness in young people.

Informants reported that all these problems had increased since the tensions. Furthermore, they led to other problems causing individuals to "worry too much" and making "a bad community". In the wake of the conflict, people had experienced increased economic hardship, social dysfunction and stress. A woman stated: "Not one thing but lots of small things coming on top of each other." Women mentioned they had become concerned about safety, both moving around the local area and travelling to Honiara after the road was reopened. One told us "before the tensions our memory was free but afterwards our memory has fear". The women also worry about what will happen when the foreign police and soldiers who make up RAMSI leave; thus fearful memories are compounded by fear for the future.

Contrary to our expectations, conflict-related trauma conditions, although acknowledged to exist, were not identified as major problems. For help, community informants said that people should see the trauma support workers for counselling.

**Figure 3:**
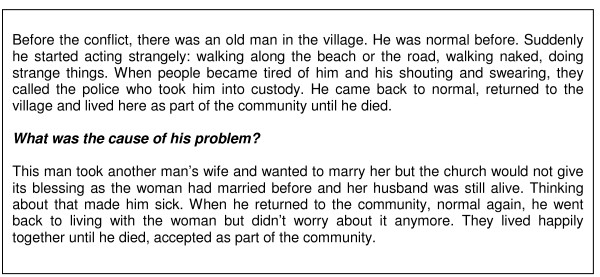
**Mental disorder due to conflict with church teachings**. Case related by older woman, edited and paraphrased for clarity.

One man was particularly concerned about conflict between accepted cultural practices and church teachings, citing the example of a married person who had left their spouse to live with someone else and therefore "cannot fully enter into the sacrament of the church". An example of how this was perceived to have led to mental illness is given in Figure 3.

### Community understanding of mental health and mental problems

When asked what "mental health" means, our informants told us that that it had to do with thinking and behaviour or action, and to do with the brain. In Solomon Islands Pijin, the word "mentol" (mental) is generally used to refer to an individual who is crazy or mad while "krangge" is the commonly used term for mental illness. In the local language of the area, people use "bule" for mental illness and "melu" to describe someone who is withdrawn and crying. "Bubuleha" means simple or stupid – "not really 100% in their thinking".

If a person thinks and behaves properly in the community, he or she is considered mentally healthy. Conversely, not behaving properly in the community is taken as a sign that someone has mental problems. An older woman remarked on the connection between mental health problems and excessive drinking: "It causes them to lose control and [to have] loose thinking." Examples of mental problems elicited included: antisocial behaviour (such as swearing, shouting and damaging property); fighting; just sitting, mind wandering, not concentrating; learning problems among students; and "something bad from the past that continues to exist in the mind".

**Figure 4:**
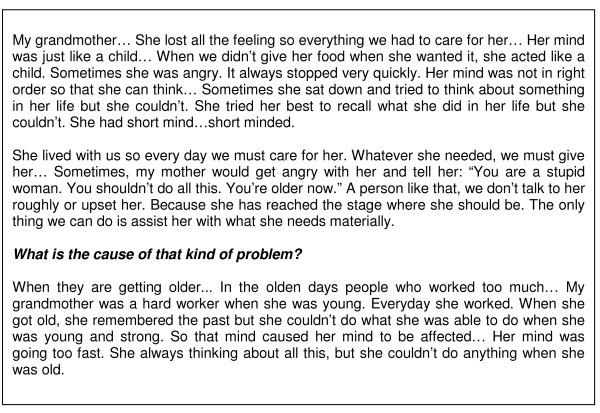
**Mental disorder in old age**. Case related by kastom doctor, edited and paraphrased for clarity.

Some respondents were able to describe a case or knew of someone who had severe mental illness. When this person caused a problem to the community, he or she would be sent to Kilu'ufi (the national mental hospital located on the island of Malaita). On their return to the community, there was no formal follow-up care. Family members helped as well they could: "We go out and talk to him, ask him what he needs." Respondents noted that sometimes relatives do not feed these people properly. Other people often make jokes and tease the person. They are neglected by the chiefs and government. One elder told us "They are lost in society". Figure 4 describes the circumstances of one old woman with cognitive and emotional problems.

The majority of people interviewed – community leaders and members – had little experience with mental health services and limited knowledge of the potential contribution of modern medicine or psychiatrists (specialist doctors in mental illness) in treating people with mental problems. One of the village chiefs told us: "I did not see any mental person being cured by modern medicine". Another elder observed that, if such treatment were available, affected persons should have been referred for help and "cured already". For most people, mental health services meant "Kilu'ufi", the national psychiatric unit in Solomon Islands. Few had any understanding about the type of treatment provided. Only two informants knew that there was a community-based mental health service in Honiara. One of them, a kastom doctor, reported that he had helped some mentally ill people to get to the general hospital in Honiara where they were treated and recovered. No-one knew how to refer someone to the mental health service.

### Help seeking for mental problems

Respondents reported that initially, people with mental problems turn to family and friends. Relatives may provide advice, assist with communication (e.g. by talking to a young girl's parents on her behalf) or encourage further help seeking. After that, people turn to others based on their understanding of the nature of the problem and its causes. If it is a kastom problem, people look to the chief for help; if it is a problem they think the church can deal with, they turn to the priest, deacon or catechist. For some problems (e.g. fighting and swearing) the person might seek help from both the chief and the priest. In such cases, the priest's role is to bring the parties together, to provide advice and facilitate mediation and reconciliation. The chief, on the other hand, will evaluate the problem and specify the amount of compensation. Both activities – reconciliation and compensation – are considered necessary for a successful outcome. Other community resources that people turn to include the trauma support workers and kastom doctors. Sometimes they turn to the clinic or the health service. Rarely, if someone is very disruptive, the police are called to take the person into custody.

The trauma support program was well known in some villages and less so in others. There were two experienced trauma support workers in the community. In addition, the priest and 32 catechists had received basic training in communication (listening skills, questioning and body language) through the program.

**Figure 5:**
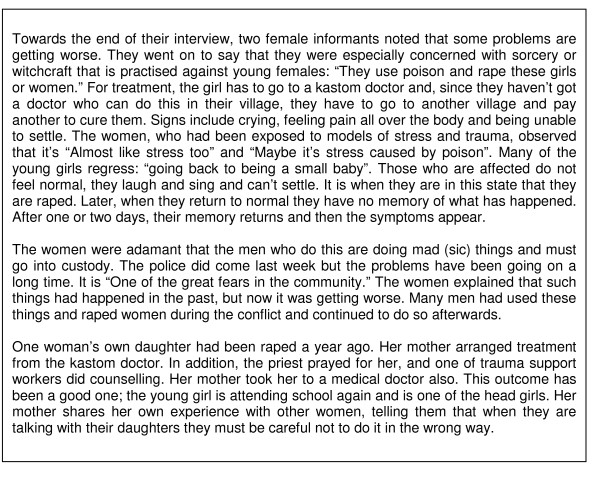
**Mental disorder attributed to supernatural causes**. Concerns expressed by two older women, edited and paraphrased for clarity.

Kastom medicine involving the use of plants and tree bark was mainly employed in the treatment of physical problems or illness. However, people believed that kastom doctors could also cure mental disorders that had their roots in kastom or the supernatural – sorcery, witchcraft or ancestor spirits (Figure 5). The nurses in the clinic mostly consulted with patients who had headaches, body pain, fever, cough, diarrhoea and skin sores; treating everybody from babies to old people. Occasionally, they referred someone with a mental problem to the doctor and the mental health service in Honiara, or to one of the trauma support workers in the community.

Certain individuals possessed knowledge and skills from more than one healing tradition. One of the trauma support workers explained that she uses three approaches with women, who often approach her when there are arguments within the family: counselling; kastom if the woman is physically sick; and prayer, often involving the husband. If she thinks the woman needs western medicine, she advises her to go to the local clinic.

Excessive use of alcohol and marijuana present a special challenge. These are relatively new problems and there are no established traditional responses at the community or individual level. Respondents were aware of, but unsure about, the potential role of health services or police in addressing these problems. One woman suggested that, for the person experiencing drug-related problems, the only answer was to stop: "If he eats good food like vegetables that will help him. Maybe the doctor has some type of medicine to drain him out... But the only thing is to stop. Somebody should advise him of that. 'Oh, you stop'... Everyday comfort him and care for him...Then he must look after (himself)...Must not smoke."

### Roles of different people and organisations

Informants saw clear and complementary roles for community members, community leaders, churches, NGOs and the government across the spectrum of interventions: from mental health promotion to prevention, early intervention and care for those with problems. They emphasised the need for a collaborative approach; for the local chiefs and other groups to work together to help vulnerable individuals. Talking about people with mental illness specifically, one man observed: "In this community itself I see some sign of hope that we are doing something here ... But in some communities around here I see that when someone is like that they neglect them, and they are scared of them, and they don't say good things about them, and they don't care for them."

#### Family and community

Harmonious family life was considered essential. Parents were expected to be role models, with responsibility for fostering good attitudes and behaviours in children (e.g. participating in church activities) and discouraging bad attitudes or activities that will bring problems to the family or community (e.g. using drugs). The community can assist by giving advice and visiting families where there are problems such as domestic violence.

#### Chiefs and kastom doctors

Our discussions revealed that chiefs and kastom doctors have a key responsibility in helping to make sure that community members live a happy life free from sickness, fighting and other problems. They are expected to work together, and with other community leaders and parents, so that problems in the community are solved appropriately and quickly. In living up to their position and authority, they ensure continued respect for their roles and contribute to maintenance of traditional culture. Kastom doctors have a role in curing mental disorders related to kastom.

#### Churches and non-government organisations

Our informants said that the church had a number of roles and that church leaders were also role models. Priests and catechists should work with the trauma support program, pray for people who have mental problems and involve them in church activities. They should encourage families in their congregation to be actively involved in church activities and young people to take up the ministry. Suggestions to engage young people, and keep them busy and away from trouble, included bible-sharing, youth groups, sports, and community programs such as visiting old people.

Respondents said that the NGOs should visit the communities and help them address pressing issues such as marijuana use and drinking, and "how to make good families". In addition, different NGOs could play a role in assisting the community to promote sports or helping the government to finance small projects for young men and women.

#### Government services

On the whole, respondents were unhappy with the level of government services and frustrated that issues affecting the people of the community had not been addressed. They wanted government support to start small income-generating projects that would keep young people busy and community awareness programs about drugs and other problems. In particular, respondents said the health service should provide or facilitate more health talks on the use of marijuana and other topics, including ways of helping people with severe mental illness. As indicated above, there was little knowledge about available services.

### Mental health, peace and conflict

Respondents saw a link between mental health and peace or conflict, with people in good mental health being more able to avoid trouble, to come together to solve problems, thus leading to peace. In contrast, when a person's mind is disturbed it is difficult to make good decisions and easy to get into conflict. Feelings of anger and a desire for revenge contribute to conflict within families and communities and at the national level. One chief spoke of how people with mental illness may be exploited; pointing out that sometimes individuals with "clear thinking" plan bad things and ask others "whose mind is disturbed" to carry them out. He believed that those in good mental health should give good advice that can bring peace.

## Discussion

Overall, the willingness and openness of the informants to share these insights and opinions seemed to be strengthened by their understanding that their input would contribute to, and possibly lead to improvements in, policy development and service delivery. The involvement of representatives from a rural community (three women and four men) in a national workshop was unprecedented in the Solomon Islands and proved to be enlightening for all participants as well as empowering for these seven community members.

Low and middle income countries around the world, especially states exhibiting a high degree of 'fragility', face numerous challenges in promoting the achievement of the Millennium Development Goals (MDGs) and securing health gains. The focus on child and maternal health, and on infectious diseases, indirectly undermines the priority placed upon other important areas of health promotion and health systems development, such as mental health. Indeed, although not recognised in the MDGs, mental health is itself increasingly recognised as central to broader development activity and to securing the health and stability required for development to proceed[13]. The links between mental health and physical health, and between mental health and healthy lifestyles, the ability to care for one's children, to maintain employment and to contribute to social cohesion and community development, deserve greater attention. As Gureje and Jenkins note: "individual and population interventions that improve an individual's mental health will enhance the individual attributes necessary for constructive social interaction and for the assumption of a productive social role". Building on community understandings and practices is crucial to unleashing the potential for evolving mental health systems and services to play their part in promoting broader health and development.

### Problems identified

The main problems identified by the community were consistent with the limited earlier research in the Solomon Islands. A national village survey, involving focus groups with men, women and youth, undertaken in late 2005, found that the top four social problems were alcohol, marijuana, domestic violence and teenage pregnancy[14]. A nation-wide study into the needs and priorities of young people using participatory methods identified the following common issues: lack of community participation, aimlessness, poverty, drug and alcohol abuse, teenage pregnancy, illiteracy and inadequate youth activities[15].

While epidemiological data are lacking, a number of reports suggest that pervasive use of tobacco and psychoactive substances is becoming an increasing problem in the Solomons[9,15,16]. Since World War II, a traditional betel chewing community has been transformed into a society with multiple substance use and abuse. In the present study, smoking marijuana was regarded as the major cause of psychosis. Alcohol intoxication was associated with violence, including domestic violence. Violence has a significant adverse impact on women's mental health[17].

Our informants recognised a range of mental problems, from transient stress and episodic disturbances to chronic disabling disorders. They ascribed these to a range of causes – social, psychological, biological and supernatural. Many were aware of someone who suffered from severe mental illness. There was, however, a dearth of knowledge about the potential for effective treatment, the mental health service or how to access it.

The finding that abnormal behaviour was often attributed to the supernatural is in contrast to a study among market vendors, white-collar workers and peri-urban dwellers in Suva, the capital of Fiji, admittedly a neighbouring island country subject to much greater Western influence over the past century. In the Suva study, 86% knew that there were many types of mental disorders, 63% knew that the hospital offered mental treatment, 54% acknowledged the efficacy of medications, and witchcraft was not considered a cause of mental illness. In that study it was found that the higher the level of education, the greater the knowledge of mental illness[18]. In the Solomon Islands, where the majority of students do not study beyond primary school[19], mental health service providers that we interviewed identified lack of education and persistence of traditional illness beliefs as a major barrier to service utilisation.

### Help seeking and local resources

For assistance with mental and social problems, community members turn first to family and friends and then to other community resources. Often, more than one avenue is pursued – simultaneously or sequentially. If the intervention or treatment fails and the problem is not resolved, sufferers move up the hierarchy of interventions. Many of the accounts elicited suggested that, as they do so, individuals adapt their beliefs accordingly. This leads us to agree with Williams & Healy[20] who have argued that the term 'explanatory model'[21] does not fully convey the plasticity of beliefs in such circumstances, and have proposed an 'exploratory map' as a replacement.

The term 'counselling' also deserves consideration. Models of counselling developed in the West, based on western theories, usually focus on individual needs and responses and overlook the family and the broader social context. The term was widely employed by respondents who saw the value of talking problems through with others. As used, it covered a range of activities (mostly involving individuals and, less often, couples) including listening, reassuring, explaining, giving advice, problem solving or teaching relaxation. This community had been exposed to models of pastoral counselling as well as to the trauma support program. There was a strong overlap between the two.

### Implications for policy and practice

The Solomon Islands, like other Pacific island countries, is undergoing a series of profound societal shifts[19,22]. In addition, in the Solomons armed conflict has further disrupted society and has added another layer of insecurity and fear, especially for women. This community-level research revealed how psychosocial and mental problems affect the whole community. Perhaps more importantly, we learned of the various ways that the community is responding to these societal stresses and their sequelae, and the importance to them of being able to seek help from *both *traditional sources and any new services and programs available.

Previous qualitative research on health seeking behaviour in the Solomons has highlighted medical pluralism, with kastom medicine operating alongside biomedicine[10]. This research, with its focus on mental and psychosocial problems, also highlighted the role of church leaders and NGO workers. Like the kastom doctors, they are in a good position to identify people with chronic or severe mental problems and refer them to the health service. However, first these community leaders need to be trained, and communication paths and referral protocols established. Other leaders, such as chiefs, elders and teachers, should also be involved in any training, which should include regular refresher sessions as well as an initial workshop. The nurses and nurse aides at the clinic need to be skilled up to provide a primary care response (basic assessment and treatment) and to refer to mental health service when necessary. Subsequently, the local health workers will have an important role in follow-up and review. The relevant community responses should also be pursued, including the processes of compensation and reconciliation. These are an essential part of holistic care in the Solomon Islands context.

Traditional healing practices vary across the country; they include song and dance, as well as food and kastom medicine. Further investigation is needed on ways in which traditional healers could be linked with mental health services.

The lack of penetration of mental health services into this rural, but hardly remote, area is of concern. The community findings offer strong support for the two key strategic directions of the WHO's Western Pacific Regional Strategy for Mental Health[23]: an intersectoral approach to mental health promotion and the prevention of mental illness; and the integration of treatment for mental disorders into general health services and a more informed understanding of mental health in the wider community. It is notable that these two strategies are seen as separate but complementary.

Since respondents were eager to receive information about mental and substance-related disorders and learn what assistance was available, there could be value in using participatory techniques including role play, story-telling and drama, rather than outmoded yet frequently employed didactic approaches [15]. Participatory methods and use of story, part of the traditional Pacific style of education for health, are again being welcomed[24]. It is important to ensure inclusion of those who may not usually participate such as single mothers and rebel groups[25].

Improving the status and safety of women is central to development in the Solomon Islands [19]. Tackling the problem of gender-based violence and the consequent increased rates of psychological disorder among women should be part of a public health strategy[17]. The special issues of youth, the group most vulnerable to mental disorders[26], also need to be addressed. Mental health promotion should take a life span approach, addressing the needs of children, youth, adults and older people[27].

### Limitations

We selected a district on Guadalcanal anticipating it to be illustrative of the types of challenges facing communities and service providers. We appreciate that every community will be in many ways unique, especially given the huge geographic and ethnic diversity in the Solomon Islands. Indeed we found that even within this one small area, differences were observed in patterns of interaction with people and organisations beyond village boundaries. Given limited time and resources, we did not seek to undertake a detailed ethnography; our purpose was rather to gain some insight into the range of experiences likely to be occurring in any local setting. By employing a local researcher and with the advice and support of local networks we were able to achieve such community insights. Overall, whilst we recognise our results are not generalisable beyond this community, there are valuable implications that may have relevance for similar populations.

### Further research

Unfortunately, no epidemiological data on mental disorders and psychosocial problems is available in the Solomon Islands, as is the case in almost all Pacific countries. Without knowledge of the extent of the problems, it is impossible to set goals and targets for interventions. Both quantitative and qualitative data are needed, collected by research teams including international and local researchers. Advisory committees should include members of government departments and NGOs working in the area, as well as representatives of the local communities.

## Conclusion

In the rural community studied, mental health is viewed as an integral part of health and wellbeing. Mental and psychosocial problems cause distress to individuals and families and adversely affect the community. The mental health service was largely unknown apart from the inpatient facility. There was only one NGO program with an ongoing presence. Without access to specialist services and support, community leaders and members struggled to address increasing problems as best they could. In particular, severe mental disorders and the emerging problems of substance abuse exceeded their capacity. Left untreated and unchecked, such problems will further undermine social cohesion and mental health and wellbeing, increasing the likelihood of future instability and conflict.

These findings underscore the need to extend mental health care beyond the capital city and other urban centres to rural villages where most of the people live. In a low-income country with a challenging geography like the Solomon Islands this is best done by developing a community-centred mental health service, integrating mental health into primary health care and promoting intersectoral collaboration. There is a need for innovative approaches that utilise community structures, churches and NGOs to reach a dispersed and linguistically diverse population. Community participation is essential, with young people, women and men contributing at national, provincial and district levels.

Evolving mental health systems are able to expand care through engagement with traditional and other community structures. Better understanding of community perspectives and approaches allows services to be enhanced and extended, an enabling policy environment created, and mental health to be more actively promoted.

Our insights from one post-conflict fragile country in the Pacific, are offered as a stimulus to a more holistic population approach: joining up the fragmented emphases on the MDGs with those focused on other pressing health problems such as mental health, recognising the potential value of both traditional and biomedical approaches, promotion, prevention and care, and the importance of ensuring coherence between policy, services and community experience.

## Consent

Written informed consent was obtained from the participants for publication of this manuscript.

## Competing interests

The authors declare that they have no competing interests.

## Authors' contributions

IB contributed to the study's conception, was deeply involved in data collection, management and analysis and was the primary author of this paper. ABB and JR contributed to the study's conception, assisted in data management and analysis and played a part in the writing of the paper. DS contributed to the study's conception and critically reviewed the paper at various stages in its evolution. ABZ contributed to the study's conception as the chief investigator and played a part in the writing of this paper
